# Supplementary material for: Ceramic resonators for targeted clinical magnetic resonance imaging of the breast
Source: Nat Commun. 2020 Jul 31;11:3840. doi: 10.1038/s41467-020-17598-3 (PMC7395080; doi:10.1038/s41467-020-17598-3)
Supplement: Supplementary file 1 — Supplementary Information [file 41467_2020_17598_MOESM1_ESM.pdf]

Supplementary Information

Ceramic resonators for targeted clinical magnetic resonance imaging of the breast

A. Shchelokova *et al.*

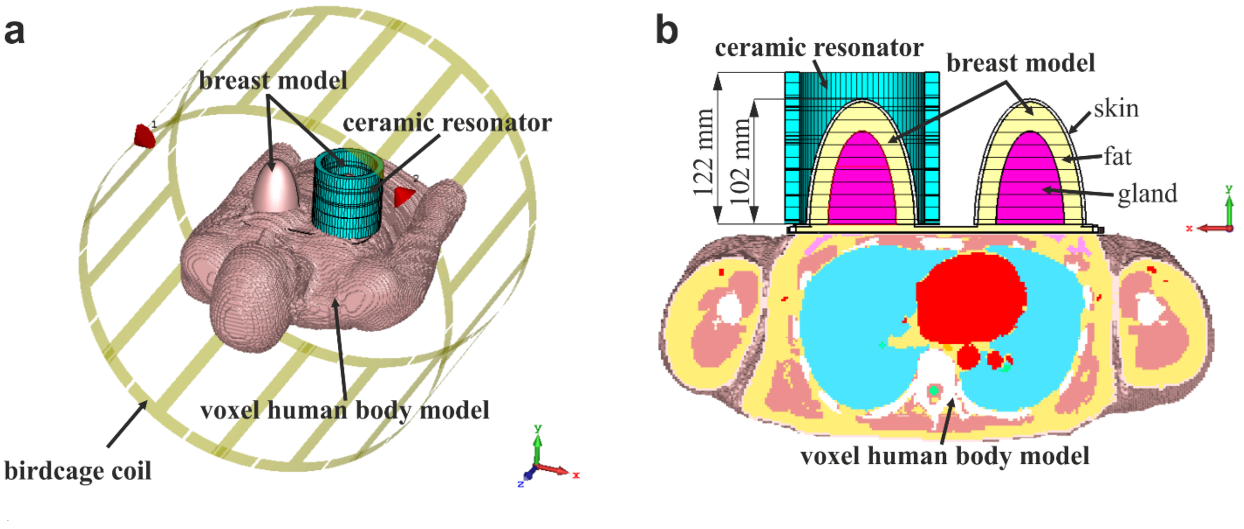

Supplementary Figure 1. General view of the proposed setup. (a) the ceramic resonator is placed around the target area (right breast). (b) Axial slice of the voxel human body model with the created human breast model and dielectric resonator.

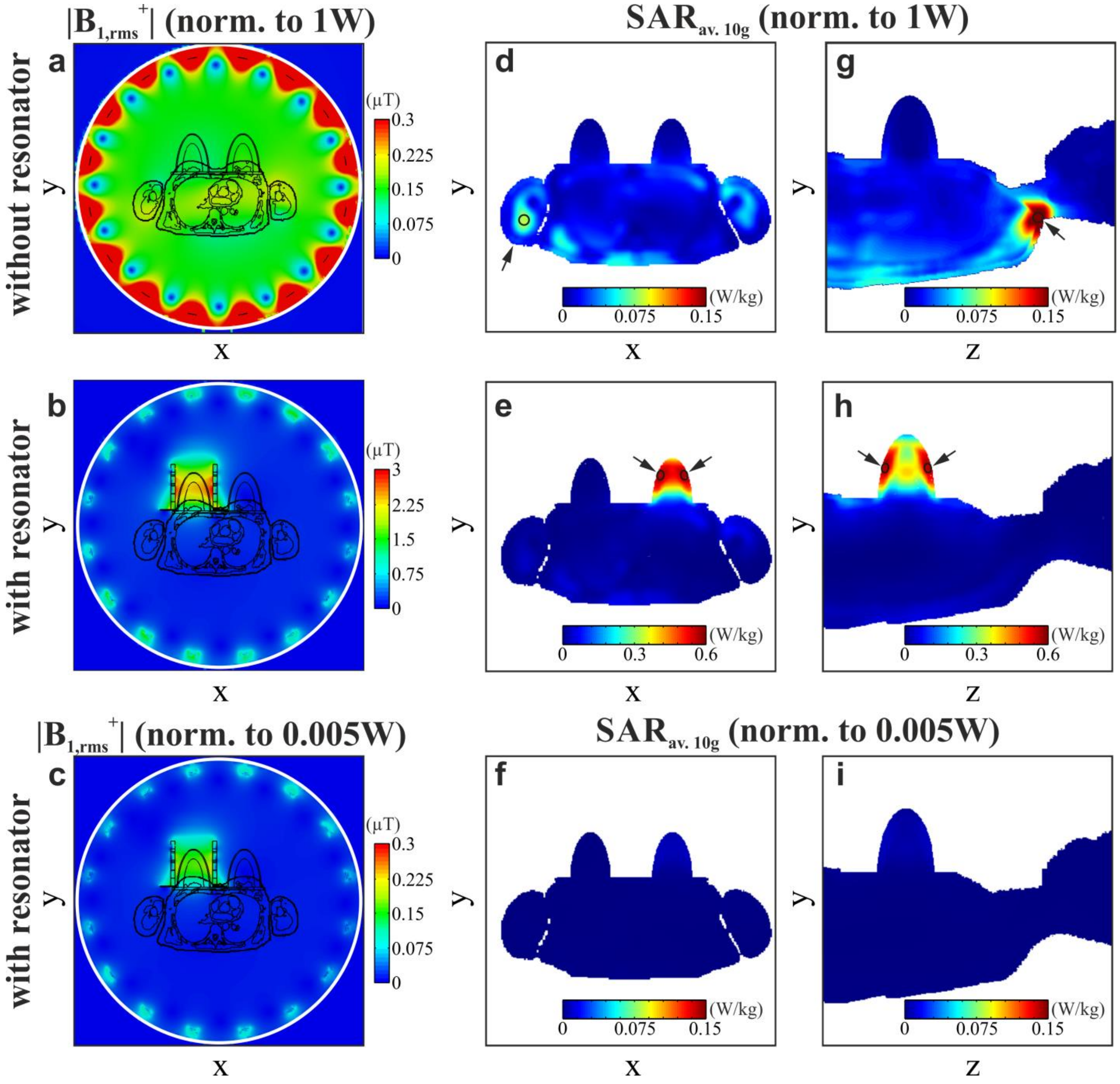

Supplementary Figure 2. Numerical simulation results of the $|B_{1,rms}^{+}|$-field, and $SAR_{av.10g}$ distributions for a human voxel model placed inside the birdcage body coil. The calculated $|B_{1,rms}^{+}|$ maps without (a), and with (b) the resonator for 1 W of total excitation power, and (c) with the resonator for 0.005 W, to create the same mean value of $|B_{1,rms}^{+}|$ in the breast as for the reference case (the birdcage body coil alone). The calculated $SAR_{av.10g}$ maps: (d,g)—reference case without the resonator for 1 W of accepted power ($|B_{1,rms}^{+}|$ =0.14 uT in the breast area); (e),(h)—with the resonator for the same accepted power 1 W as in the reference case ($|B_{1,rms}^{+}|$=2 uT); (f),(i)—with the resonator for the same $|B_{1,rms}^{+}|$ as in the reference case (accepted power 0.005 W). White solid lines indicate the boundaries of the birdcage coil. Black circles and arrows depict peak spatial SAR regions.

Supplementary Table 1 | The comparison of the P_{acc} and corresponding mean B_1^+ values across the breast area for normal and the first-level controlled operating modes of an MRI examination without and with the resonator in place.

Operating mode		Without resonator	With resonator
Normal ($SAR_{av.10g}$=10w/kg)	P_{acc}^*	66 W	17 W
	mean $B^+_{1,rms}$ in the breast	1 uT	8 uT
First-level ($SAR_{av.10g}$=20w/kg)	P_{acc}^*	132 W	34 W
	mean $B^+_{1,rms}$ in the breast	1.5 uT	11.7 uT

$^*P_{acc}$ – power accepted by the system.

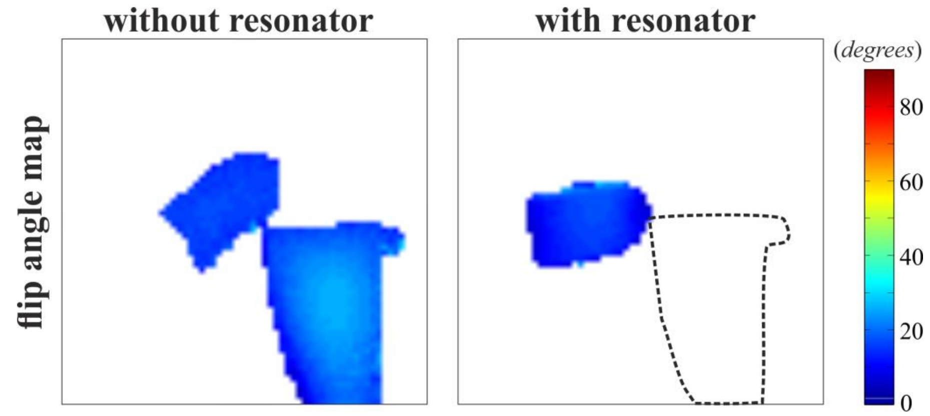

Supplementary Figure 3. The flip angle maps created by the birdcage coil without and with the dielectric resonator. The maps were produced from sagittal GRE images using a double flip angle method. The small liquid phantom in the center of the resonator. The black dashed lines indicate the boundaries of the big phantom, which was imitating the body and is not presented on the right map since it produced no signal.

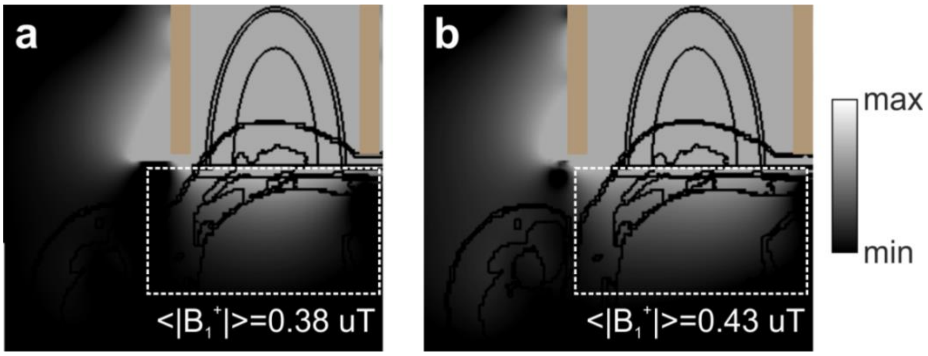

Supplementary Figure 4. Numerical simulation results of the transmitting magnetic field (B_1^+) for (a) the proposed ceramic resonator and (b) the resonator with a 20% larger inner diameter. Brown shaded areas show the boundaries of the resonator. White dashed lines indicate the field-of-view improvement of the lateral part, where the mean value of the B_1^+-field was calculated.
